# Corrigendum to “Prime-Boost Vaccination Using Chemokine-Fused gp120 DNA and HIV Envelope Peptides Activates Both Immediate and Long-Term Memory Cellular Responses in Rhesus Macaques”

**DOI:** 10.1155/2020/5471638

**Published:** 2020-10-29

**Authors:** Hong Qin, Pramod N. Nehete, Hong He, Bharti Nehete, Stephanie Buchl, Soung-chul Cha, Jagannadha K. Sastry, Larry W. Kwak

**Affiliations:** ^1^Department of Lymphoma and Myeloma, M. D. Anderson Cancer Center, The University of Texas, Houston, TX 77030, USA; ^2^Center for Cancer Immunology Research, M. D. Anderson Cancer Center, The University of Texas, Houston, TX 77030, USA; ^3^Department of Veterinary Sciences, M. D. Anderson Cancer Center, The University of Texas, Bastrop, TX 78602, USA; ^4^Department of Immunology, M. D. Anderson Cancer Center, The University of Texas, Houston, TX 77030, USA

In the article titled “Prime-Boost Vaccination Using Chemokine-Fused gp120 DNA and HIV Envelope Peptides Activates Both Immediate and Long-Term Memory Cellular Responses in Rhesus Macaques” [1] (published when the journal was titled *Journal of Biomedicine and Biotechnology*), as raised on PubPeer, there is duplication of panels in Figure 2 [2].

Figure 2(e) is the same as Figure 2(i).

Figure 2(f) is the same as Figure 2(i), though Figure 2(f) has a blue block in the top right of the dot plot. Figure 2(g) is the same as Figure 2(k).

Figures 2(e)–2(g) were directly above Figures 2(i)–2(k) in the figure. Dr. Jagannadha Sastry reexamined the original data with the third author Hong He who conducted the assay and data analysis and found that this error was introduced when copying data from FlowJo to PowerPoint. The authors provided a corrected figure redone by Dr. He, as shown below:

## Figures and Tables

**Figure 1:**
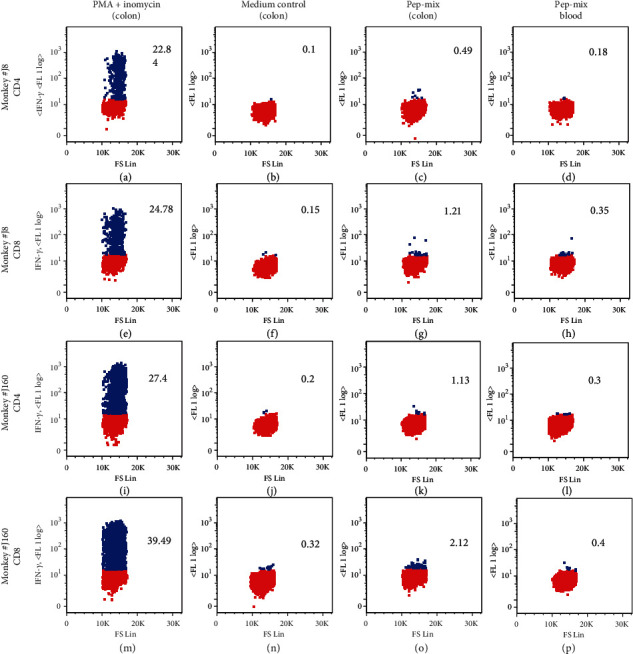
The immunization strategy elicited mucosal long-term memory T cell immune responses. Production of IFN-𝛾 by CD3^+^CD4^+^ or CD3^+^CD8^+^ memory T cells isolated from the colon was analyzed in the vaccinated macaques one year after final peptide-cocktail boost. Lamina propria lymphocytes (LPL) from colon biopsy samples were stimulated with peptide-mix or mitogens for 6 h. Both untreated (control) and stimulated cells were stained for surface markers, followed by fixation, permeabilization, and intracellular staining of IFN-𝛾. Live cells were identified by gating on Aqua-negative cells. The cells gated on CD3^+^CD4^+^ and CD3^+^CD8^+^ were further separated as the memory population according to the expression of CD95 (data not shown). The percentage values indicate the population of IFN-𝛾—producing CD3^+^ CD95^+^CD4^+^ or CD3^+^ CD95^+^CD8^+^ lymphocytes.
